# Horse allergen sensitivity and respiratory symptoms among horse farm workers

**DOI:** 10.3906/sag-1912-39

**Published:** 2020-06-23

**Authors:** Burcu BARAN KETENCİOĞLU, İnsu YILMAZ, Nuri TUTAR, İnci GÜLMEZ, Fatma Sema OYMAK

**Affiliations:** 1 Department of Chest Diseases, School of Medicine, Erciyes University, Kayseri Turkey; 2 Division of Immunology and Allergy, Department of Chest Diseases, School of Medicine, Erciyes University, Kayseri Turkey

**Keywords:** Horse farm workers, horse allergen, occupational exposure, respiratory symptoms, skin prick test

## Abstract

**Background/aim:**

Sensitivity and symptoms related to animal proteins have been investigated in various occupational groups. However, data from horse farm workers are limited. We aimed to determine horse allergen sensitivity in the horse farm workers, and to evaluate its relationship with respiratory symptoms and functional parameters.

**Materials and methods:**

A total of 110 subjects were enrolled in the study. The study group consisted of 80 horse farm workers. Face-to-face surveys, skin prick tests (SPT), and pulmonary function tests (PFT) were performed in the study group. Control group consisted of 30 healthy subjects. SPT and PFTs were also performed for control group. The SPT test results of the horse farm workers were compared with the SPT results provided from the medical records of 1376 subjects who admitted to the outpatient clinic with respiratory symptoms.

**Results:**

Atopy rate was significantly higher in horse farm workers than in healthy subjects (41% and 13%, respectively; P = 0.008). Horse allergen sensitivity was positive 8/80 (10%) in horse farm workers, 0/30 in healthy subjects, and 32/1376 (2%) in medical records of subjects who were admitted to the outpatient clinic with respiratory symptoms. (P = 0.07, P = 0.001, respectively). There was no statistically significant relationship between respiratory symptoms and horse allergen sensitivity in horse farm workers (P = 0.67). However, mean FEV1 ratios were lower in horse farm workers with horse allergen sensitivity than healthy subjects (88.6% ± 17.9, 103.7 ± 10, P = 0.031, respectively).

**Conclusion:**

Atopy and animal allergen sensitization were significantly higher in horse farm workers, suggesting the relationship between the intensity of specific allergen exposure and the sensitization to this specific allergen.

## 1. Introduction

Animal proteins are known to be antigenic structures with high allergenicity. Continuous and intense exposure to animal allergens can cause sensitization to these allergenic proteins for individuals with genetic predisposition [1–3] and can lead to various clinical conditions from urticaria to respiratory distress [4].

There are various data about sensitization to animal proteins in occupational groups such as veterinarians, animal laboratory personnel, and farmers [5,6]. In a study that evaluated veterinarians in California, it was reported that 40% of the cases described respiratory and/or skin symptoms related to specific animal contact, and that cats and dogs were the animals that caused these symptoms most frequently [7]. In another study, asthma prevalence in veterinarians was found to be three times higher than in the control group [8]. 

There are also studies where horse allergen sensitivity in the population was evaluated without occupational exposure [4,9,10]. In a study conducted in the Naples region of Italy, horse allergen sensitivity in the local community, without occupational exposure, was found to be 3.43% [11]. In another multicenter Italian study, horse allergen sensitivity was determined to be 5.32% among patients seen at an allergy outpatient clinic [12]. 

However, there are a limited number of studies evaluating respiratory symptoms and horse allergen sensitization rates in people working at horse farms, which have been increasing in numbers in recent years. In a study conducted by Tutluoglu et al., the authors evaluated the frequency of horse allergen sensitivity and respiratory symptoms of the Veliefendi Hippodrome workers and horse riders in Istanbul. Horse allergen sensitivity was 3.75 times higher than in the control group. However, no comparison was made with horse allergen sensitivity rates in the general population [13]. 

Therefore, the aim of this study was to determine atopy prevalence and horse allergen sensitivity rates in horse farm workers within our region, and to compare these values against the sensitivity ratios of the normal population. Another aim was to evaluate the relationship between sensitivity to horse hair allergens and respiratory symptoms and to determine its effects on respiratory functions.

## 2. Methods

### 2.1. Patients

A total of 110 subjects were enrolled in the study. Four horse farms in Kayseri Province were visited between August 2015 and November 2015. The study group consisted of 80 horse farm workers (67 male, 13 female, 33.9 ± 11.8 years). The control group consisted of 30 healthy subjects (21 male, 9 female, 35 ± 12.7 years) who were older than 18 years of age and had no known chronic disease, atopy history, or occupational horse allergen exposure. The SPT test results of the horse farm workers were compared with the SPT results provided from the medical records of 1376 subjects who were admitted to the Erciyes University Pulmonary Diseases, Allergy and Immunology Outpatient Clinic with rhinitis and/or asthma symptoms between June 2014 and July 2015. The study protocol is shown in Figure. The approval of the Erciyes University Medical Faculty Ethics Committee was obtained (Date: 28.08.2015; Decision No: 2015/407).

**Figure:**
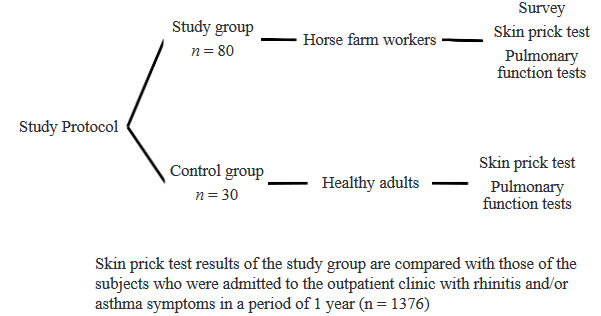
Study protocol.

Surveys were completed with horse farm workers through face-to-face interviews. The survey inquired about the job history of the workers (their jobs at their workplace, employment duration, full-time and part-time employment status), allergic disease in the family history, respiratory system symptoms, rhinitis and conjunctivitis symptoms, atopy symptoms, the relationship of these symptoms with their work and their onset time, smoking history (still smoking, never smoked, and ex-smoker, pack-year) and the presence of chronic disease. 

The SPT data of the 1376 subjects were provided retrospectively from the patients’ medical records. Demographic data of the patients were not evaluated. Demographic data, SPT results, and PFTs were also evaluated in the control group and these results were compared with the results of the study groups.

### 2.2. Skin prick test

After drops of allergen extracts were applied on the forearm of the patient, the epidermis was pricked with a prick lancet (Heinz Herenz Hamburg, Germany) and was left for 20 min. Allergen extracts were applied at a distance greater than 2 cm between each of them. Normal saline solution was used as a negative control and histamine for a positive control. At the end of a 20-min period, an induration size that was ≥3 mm larger than the size of the induration resulting from a negative control was considered positive. Positivity against any of the common aeroallergens in SPT was considered as atopy.

The skin prick test was performed in all groups with a standard aeroallergen panel [*D. Farinae*, *D. Pteronyssinus*,* Betulacees*,* Salicacees*, grasses mixture, *Compositae*, trees mixture, cereals mix, *Penicillium mix*, *Germanica*, *Aspergillus mix*, *Cladosporium*, *Alterneria*, cat, dog, and horse] (Stallergenes, France) and by an experienced nurse and doctor at the clinic.

### 2.3. Pulmonary function tests

PFTs of the study group and control group were performed by using a portable Nspire KoKo Legend 314000 device that is registered to the Pulmonary Diseases Department of the Medical Faculty at Erciyes University. PFTs were performed in a seated position and with a soft nose clip. At least three measurements were taken for each individual and the best values were recorded. FEV1 (Forced expiratory volume in 1 s), FVC (Forced vital capacity), and FEV1/FVC values were measured. Values were presented as the percentage of the expected value of the corresponding age and height. 

### 2.4. Statistical analysis

Data was analyzed by using the IBM SPSS Statistics 22.0 (IBM Corp. Armonk, New York, USA) statistics package software. As descriptive statistics, values were represented as a number of units (*n*), percentage (%), mean ± standard deviation (*x̄* ± ss), and median (min-max). The normal distribution of the data of numeric variables was analyzed by using the Shapiro–Wilk normality test and *Q*-*Q*graphs. The Mann–Whitney* U* test was used for variables with nonnormally distributed two-group comparisons and the Kruskal–Wallis analysis was used for comparisons with more than two groups. If a difference was detected in the Kruskal–Wall analysis, the Dunn–Bonferroni test was used as a multiple comparison test. The relationship between categorical variables was analyzed with the exact method of the chi-squared test. P < 0.05 was considered statistically significant.

## 3. Results

There were 80 subjects in the study group. The study group consisted of horse riders (n = 49, 61%) and other department workers (n = 31, 39%) such as waiters, kitchen staff, cleaning staff, information desk, accounting, and administrative personnel. Demographic characteristics, SPT and PFT results of the groups are shown in Table 1. 

**Table 1:** Comparison of demographic characteristics and pulmonary function tests between the study group and control group.

	Study group (n = 80)	Control group(n = 30)	P-value
Sex, male/female, n	67/13	21/9	0.12
Age, years, mean (SD)	33.9 (11.8)	35 (12.7)	0.72
Work duration, months, median (range)	24 (1–360)	N/A	
Work time, full time/part time, n	50/30	N/A	
Job, horse rider/othera, n	49/31	N/A	
Smoking history, n (%)Still smokingUsed to smoke, former smokerNever smoked	42 (52)12 (15)26 (33)	15 (50)4 (13)11 (37)	0.91
Allergic disease history, n (%)	55 (69)	N/A	
Allergic disease in family, n (%)	25 (31)	N/A	
Pulmonary function tests, mean (SD)FEV1, %FEV1/FVC, %	96.8 (14.5)90.3 (11)	103.7 (10)86.3 (7.6)	0.0220.14

N/A: Not applicablea: Waiters, kitchen staff, cleaning staff, information desk, accounting, and administrative personnel

Thirty-three (41%) of horse farm workers had atopy and the most common sensitivity was for house dust mite, grass pollen, and cat allergens [13(16%), 13(16%), 13(16%), respectively], 20 (25%) of horse farm workers were sensitive to animal allergens [cat 13 (16%), dog 10 (13%), horse 8 (10%)]. In medical records of 1376 subjects who were admitted to the outpatient clinic with respiratory symptoms, the most common sensitivity was found to grass pollens (197, 14%). Horse allergen was detected as positive in 2% of subjects who were admitted to the outpatient clinic with respiratory symptoms and 10% of horse farm workers (P = 0.001). In the control group the asymptomatic atopy rate was 13% and no horse allergen sensitivity was detected (Table 2).

**Table 2:** Skin prick test results of the study group, control group, and the subjects who were admitted to the outpatient clinic with respiratory symptoms.

SPT	Study group(n = 80)	Control group(n = 30)	P*	Outpatient clinic(n = 1376)	P**
Atopy in SPT, n (%)MiteGrass pollenAspergillus mixCatDogHorse	33 (41)13 (16)13 (16)6 (8)13 (16)10 (13)8 (10)	4 (13)3 (10)0 (0)0 (0)0 (0)1 (3)0 (0)	0.0060.310.0120.140.0120.140.07	544 (40)187 (14)197 (14)38 (3)45 (3)42 (3)32 (2)	0.070.680.620.030.0000.0000.001

SPT: Skin prick test*: P-value for the difference between the study group and the control group **: P-value for the difference between the study group and the subjects who were admitted to the outpatient clinic with respiratory symptoms

In the study group, all of the eight workers with positive horse allergen sensitivity were male, their mean age was 39.4 ± 17.4 years, and the median employment duration was 60 months. Of these workers, 4 of 8 (50%) were sensitive to cat and 6 of 8 (75%) were sensitive to dog. The atopy rate with and without horse allergen sensitivity in the study group was 7/8 (88%) and 25/72 (35%), respectively (P = 0.018). No statistically significant difference was found between horse allergen sensitivity, and age or employment duration (P = 0.39 and P = 0.08, respectively). Comparative evaluation of the workers with and without horse allergen sensitivity is given in Table 3. 

**Table 3:** Comparison of demographic characteristics, aeroallergen sensitivity, and pulmonary function tests among horse farm workers with and without horse allergen sensitivity.

Values	Horse positive (n = 8)	Horse negative(n = 72)	P-value
Sex, male/female, n	8/0	59/13	0.34
Age, years, x̄ (SD)	39.4 (17.4)	33.3 (11)	0.39
Work duration, months, median (range)	60 (2–360)	24 (1-240)	0.08
Work time, full time/part time, n	6/2	43/29	0.76
Job, horse rider/othera, n	6/2	43/29	0.64
Atopy in SPT, n (%)CatDog	7 (88)4 (50)6 (75)	25 (35)9 (12)4 (6)	0.0180.0070.000
Upper respiratory symptoms, n (%)	2 (25)	22 (31)	1.0
Lower respiratory symptoms, n (%)	1 (13)	18 (25)	0.67
Pulmonary function tests, x̄ (SD)FEV1, %FEV1/FVC, %	88.6 (17.9)98 (19.3)	99.5 (13.1)88.5 (9)	0.950.25

SPT: Skin prick testa: Waiters, kitchen staff, cleaning staff, information desk, accounting, and administrative personnel

At least one upper and lower airway symptom was described by 24/80 (30%) and 19/80 (24%) of the farm workers, respectively. The most frequently described symptom was nasal discharge (18/80, 23%). There was no statistically significant relationship between respiratory symptoms and horse allergen sensitivity (P = 0.67) (Table 3). There were 12 (15%) horse farm workers who described respiratory symptoms related to the workplace and 58% of these workers had atopy in SPT [grass pollen 4 (67%), mite 3 (25%), dog 2 (17%), cat 1 (8%), horse 0 (0%)]. 

Mean FEV1 ratios were lower in horse farm workers than in healthy subjects (96.8% ± 14.5, 103.7% ± 10, P = 0.022, respectively). FEV1 ratios were also lower in workers with horse allergen sensitivity than healthy subjects (88.6% ± 17.9, 103.7 ± 10, P = 0.031, respectively). In addition, FEV1 ratios of workers with lower airway symptoms were lower than FEV1 ratio of healthy subjects (93.2% ± 17.5, 103.7% ± 10, P = 0.023, respectively). 

## 4. Discussion

This is the first study in Turkey to investigate work-related allergic sensitization and respiratory symptoms in horse farm workers, and to compare horse allergen sensitivity rates with a normal population who already had respiratory symptoms. In this study, it was determined that atopy rate in horse farm workers was significantly higher than in the healthy subjects. In addition, horse allergen sensitivity due to occupational exposure in horse farm workers was shown to be significantly more increased than in the healthy subjects. However, no significant relationship was found between sensitivity and respiratory symptoms.

Atopy, which is considered positivity in SPT against any of the common aeroallergens, was found in approximately two-thirds of the horse farm workers. One-tenth of horse farm workers had horse allergen sensitivity and (7/8) 88% of these workers were also sensitive to other aeroallergens. This finding confirms the results of several studies. In these studies, atopy was shown to be a risk factor for sensitization to both animal allergens and other high-molecular weight allergens [13–15].

In our study, the relationship between sensitivity to horse allergen and sensitivity to other animal allergens was also evaluated. Among the workers with horse hair sensitivity, 4 of 8 (50%) had sensitivity against cat and 6 of 8 (75%) had sensitivity against dog. These values were significantly increased in comparison to the workers without horse allergen sensitivity (P = 0.007 and P = 0.000, respectively). Consistent with our study, Tutluoglu et al. also evaluated the horse allergen sensitivity of hippodrome workers and determined that the prevalence of horse allergen sensitivity was 8.95 times higher in workers with sensitivity to other animal epithelium in comparison to those without sensitivity [13]. In another study, an urban population without any direct or occupational exposure to horse allergen was evaluated and horse allergen sensitivity was found to be 3.43%. Atopy and animal epithelium sensitivity was evaluated as a risk factor for horse allergen sensitization in the same study and the authors recommended that IgE levels of individuals with such characteristics should be evaluated before they start a job that involves horse allergen exposure [11]. This situation indicates that sensitization to horse allergen and other animal allergens may be due to a cross reaction [16] against minor allergens of especially cow [17], cat [18], dog [19], rabbit [20], and rodent [21]. 

Horse allergen sensitivity in the horse farm workers (10%) was significantly higher than in healthy subjects (0%) and, 1376 subjects who were admitted to the outpatient clinic with respiratory symptoms (2%). The higher prevalence of horse allergen sensitivity observed in horse farm workers is believed to be secondary to the increased risk of specific occupational allergen sensitization due to greater exposure to occupational allergens in comparison to the normal population. Previous studies also reported an increased allergic sensitization risk against horse allergens with occupational exposure to horses [13,22,23]. In a study conducted in Iran that evaluated 42 horse riders, the rate of horse allergen sensitivity was found to be 31% [23]. In another study by Tutluoglu et al., horse allergen sensitivity was found to be 13% in hippodrome workers [13]. 

The respiratory symptoms of horse farm workers, who had horse allergen sensitivity, were not shown to increase at work. These results have led to the consideration that although horse allergen sensitivity was found to be higher in farm workers, the respiratory symptoms of these workers were not secondary to horse allergen exposure at workplace. In addition, approximately one-sixth (12/80) of the horse farm workers described workplace-related symptoms, although, interestingly, at least one of the pollen, house dust mite, cat, and dog sensitivities was detected in these workers, none of them had horse allergen sensitivity. Contrary to our study, in another study evaluating animal laboratory workers, it was observed that the group with occupational aeroallergen sensitivity had 4-fold increased asthma, rhinitis, and skin symptoms in comparison to the nonsensitive group [15]. In our study, this result may be secondary to the misleading responses by workers due to fear of losing their jobs. On the other hand, the FEV1 values of farm workers with lower airway symptoms were significantly lower than the FEV1 values of healthy subjects. Therefore, although speculative, it was thought that workplace-related symptoms could be secondary to other allergens such as pollen and mite or irritants (fur, hay, and dust etc.).

A major limitation of the study was the absence of methods like serial PEF measurement, nonspecific and specific provocation tests, which are required to make a definite diagnosis of occupational rhinitis or occupational asthma, for horse farm. However, as the main objective was to evaluate whether horse allergen sensitization and respiratory symptoms of farm workers were different from healthy subjects, the data were presented related to the present study’s objective. Another limitation was the low number of subjects in the study group. The low number of cases in the study group led to statistical calculation and interpretation limitations. However, the evaluation of SPT results provided from the medical records of subjects who were admitted to the outpatient clinic with respiratory symptoms, which had a large number of patient results, was effective for the comparison of horse allergen sensitization in rates within Kayseri and horse allergen sensitization of horse farm workers. 

In conclusion, horse allergen sensitivity was positive in one-tenth of the horse farm workers and this rate was significantly higher than the rates of patients seen in the outpatient clinic with respiratory symptoms and of healthy subjects. These results once again revealed the relationship between the intensity of specific allergen exposure and the sensitization to this specific allergen. Atopy and animal allergen sensitization were significantly higher in horse farm workers who had horse allergen sensitivity. FEV1 values of the horse farm workers were determined to be significantly lower than the control group. Therefore, before employment, evaluation of atopy for professions involving potential exposure to occupational allergens with high immunogenicity can be instructive in terms of profession selection. 

## Competing interests

The authors declare they have no conflict of interest. All authors have read and approved the final manuscript.
